# Survival in patients with monoclonal gammopathy of unknown significance after transcatheter aortic valve replacement: a global retrospective propensity-matched real-world analysis

**DOI:** 10.1186/s40959-026-00456-4

**Published:** 2026-02-20

**Authors:** Lorenz Oelschläger, Dominik Rebafka, Christian Frerker, Thomas Stiermaier, Jan Morf, Jan Vorwerk, Anna Traub, Theo Leitner, Nikolas von Bubnoff, Cyrus Khandanpour, Ingo Eitel

**Affiliations:** 1 Department of Hematology and Oncology, University Medical Center Schleswig-Holstein (UKSH) and University Cancer Center Schleswig- Holstein (UCCSH), Campus Lübeck, Lübeck, Germany; 2 Department of Cardiology (Medical Clinic II), University Heart Center Lübeck (UKSH), Campus Lübeck, Ratzeburger Allee 160, Lübeck, 23538 Germany; 3 Department of Rhythmology, University Medical Center Schleswig- Holstein (UKSH), Campus Lübeck, Lübeck, Germany

**Keywords:** MGUS, TAVI, Overall survival, Real-world data, Retrospective study

## Abstract

**Background:**

Hematopoietic clonal disorders have previously been associated with cardiac diseases. Higher incidences of aortic valve stenosis have been observed not only in clonal hematopoiesis of indeterminate potential (CHIP) but also in monoclonal gammopathy of unknown significance (MGUS). However, studies investigating the outcome after transcatheter aortic valve implantation (TAVI) in MGUS are lacking.

**Objective:**

Investigate overall survival in MGUS patients after TAVI.

**Methods:**

We used the federated real-world data platform TriNetX to identify MGUS patients undergoing TAVI. Patients were divided into cohorts based on presence of MGUS, age or comorbidities and matched using the TriNetX propensity score matching tool. Overall survival was assessed after 1, 3 and 5 years.

**Results:**

We identified 58,796 patients with TAVI procedure. Of these, 1039 patients (1.8%) were identified with co-occurring MGUS. Our study shows superior overall survival for patients without MGUS after TAVI in the overall cohort (HR:0.792 CI95% 0.657, 0.954, *p* = 0.014) and > 75 years of age (HR:0.797, CI95% 0.660, 0.961, *p* = 0.017) at the 5-year timepoint. 5-year overall survival did not differ in younger MGUS patients (< 75 years) or patients with co-occurring chronic kidney disease, chronic obstructive pulmonary disease or heart failure (*p* > 0.05).

**Conclusion:**

In this real-world analysis in patients with MGUS undergoing TAVI, MGUS was associated with inferior outcomes after TAVI, especially for older (> 75 years) patients. Five-year overall survival did not differ among patients with common comorbidities. Our results suggest that MGUS may be linked to worse overall long-term survival following TAVI and highlights the need for further interdisciplinary cardiooncology research. Given the key methodological limitations, the present results should be considered as hypothesis-generating.

## Background

 The field of cardio-oncology which investigates the intersection of pre-malignant hematological diseases and their impact on cardiovascular outcome is gaining increasing attention. Exemplary clonal hematopoiesis of indeterminate potential (CHIP) has been associated with myocardial ischemia, worse survival in atherosclerotic cardiovascular disease and inferior outcome after transcatheter aortic valve implantation (TAVI) [1–3]. TAVI has evolved as standard of care for the treatment of aortic valve stenosis in older patients, fueled by a robust evidence base of randomized, controlled trials comparing TAVI with surgical aortic valve replacement (SAVR) in patients at high risk [4] and intermediate to low surgical risk in terms of primary safety and efficacy outcomes [5]. Additionally, studies have repeatedly shown that in low surgical risk patients, the rate of the composite outcome of death, stroke, or rehospitalization at 1-year follow-up was significantly lower [6] or noninferior in terms of all-cause mortality or disabling stroke at 24 months with TAVI compared to SAVR [7].

Monoclonal gammopathy of unknown significance (MGUS) is a clonal plasma-cell disorder not considered malignant but often precedes other plasma-cell associated malignancies such as multiple myeloma (MM) or amyloidosis (AL) [8, 9]. Previous studies have shown significantly higher rates of aortic valve stenosis in patients with MGUS [10, 11], but clinical studies investigating overall survival in MGUS patients after TAVI are missing.

In this study we performed a retrospective analysis utilising the federated real-world data platform TriNetX (TriNetX, LLC, Cambridge, Mass., USA) which allows the analysis of global de-identified patient data provided by health care organisations (HCOs). Using this unique database, we examined the overall survival at different follow-up timepoints and in the context of common comorbidities for patients with MGUS after TAVI.

## Methods

We performed a retrospective data analysis using the federate research platform TriNetX. The de-identified data includes diagnoses, procedures, medications, laboratory values from electronic medical records (EMRs) provided by a global network of health care organizations (HCOs). The global collaborative network includes 146 HCOs, academic and non-academic medical centers. TriNetX harmonizes de-identified patient data to accepted standards like the international classification of diseases (ICD) [12]. Information about patient genetics is limited [13, 14]. Data was obtained from the TriNetX platform on March 5^th^, 8^th^ and 18^th^ .

### Cohort definition/patient selection

Patients were included at age 18 and above. Inclusion criteria are shown in Table 1. Patients were identified using the international classification of diseases (10^th^ revision) with the following codes for diseases and procedures: ICD-10 code D47.2 (monoclonal gammopathy of unknown significance (MGUS), ICD-10 code I.35 − 0 (non-rheumatic aortic valve stenosis (NrVS) and current procedural terminology (CPT33361: transcatheter aortic valve replacement (TAVI) with prosthetic valve replacement; percutaneous femoral artery approach). Patients with co-occurring diagnosis of MM or AL were excluded in the MGUS cohort. We established patient cohorts depending on age with either presence or absence of MGUS and/or comorbidities (Supplementary Fig. 1). Next, we compared overall survival at 1, 3, and 5 years.

**Table 1:** Inclusion/exclusion criteria and criteria for subgroups

Inclusion
monoclonal gammopathy of unknown significance (MGUS) (ICD-10-CM code D47.2)
non-rheumatic aortic valve stenosis (NrVS) (ICD-10 code I.35 − 0)
transcatheter aortic valve replacement (TAVI) with prosthetic valve replacement; with transfemoral approach (CPT 33361)
Age > 18

**Exclusion**
multiple myeloma (MM) (ICD-10-CM code C90)
amyloidosis (AL) (ICD-10-CM code E85)

**Subgrouping (inclusion criteria)**
Other chronic obstructive pulmonary disease (COPD) (ICD-10-CM code J44)
Chronic kidney disease (CKD) (ICD-10-CM code N18)
Heart failure (HF) (ICD-10-CM code I50.0)

*AL* Amyloidosis, *COPD* Chronic obstructive pulmonary disease, *CKD* Chronic kidney disease, *CPT* Current procedural terminology, *HF* Heart failure, *ICD* International classification of diseases, *MGUS* Monoclonal gammopathy of unknown significance, *MM* Multiple myeloma, *NrVS* Non-rheumatic aortic valve stenosis, *TAVI* Transcatheter aortic valve replacement

### Study design

After identifying patients undergoing TAVI for aortic valve stenosis within the TriNetX platform, patients were divided into different cohorts based on presence (MGUS group) or absence of MGUS (Non-MGUS group), age groups (</> 75 years) and comorbidities. We defined this subgroup based on the presence of chronic obstructive pulmonary disease (COPD), chronic kidney disease (CKD) and heart failure (HF) at any stage. The cohorts were matched using the TriNetX propensity score matching tool that has previously been validated [15] adjusting for age at index event, sex, race, levels of hemoglobin (Hb), N-terminal prohormone of brain natriuretic peptide (NT-pro-BNP), hemoglobin A1c (HbA1c) and co-occurring neoplasms. The diagnosis of MGUS, NrVS and TAVI defined the index event. For co-morbidities the respective comorbidity was included as a requirement for the index event. The outcomes assessed were overall survival defined by time of index event to death.

### Statistical analysis

All statistical analysis were performed using the build-in TriNetX analytic platform. Due to the platforms data protection and confidentiality terms access to individual patient data is not possible. Survival analysis was performed using the Kaplan-Meier analysis for overall survival. Follow-up was limited to a time of 1, 3 and 5 years after the index event respectively. Statistical significance between two groups was determined using the Log-Rank test. Hazard ratios (HR) with confidence intervals (CI95%) were also calculated showing the risk of a respective event for each defined cohort. A Cox proportional hazard model was used to estimate the effect of individual covariates on overall survival. According to general convention a p-value less than 0.05 was considered of statistical significance.

#### Ethical approval

This retrospective study is exempt from informed consent. The data reviewed is a secondary analysis of existing data, does not involve intervention or interaction with human subjects, and is de-identified per the de-identification standard defined in Section §164.514(a) of the US Health Insurance Portability and Accountability Act (HIPAA). The process by which the data is de-identified is attested to through a formal determination by a qualified expert as defined in Section §164.514(b)(1) of the HIPAA Privacy Rule. This formal determination by a qualified expert refreshed on December 2020. As only de-identified data is used in this study no additional Institutional Review Board approval is required. The study complies with the Declaration of Helsinki.

## Results

On March 5th of 2025 we identified over 58,796 patients who had a NrVS and received TAVI. 1039 of these patients were identified with co-occurring MGUS and absence of AL or MM (MGUS group). The characteristics of the different cohorts are shown in Table 2.

**Table 2:** Patient characteristics before propensity score matching

	**MGUS**	**Non-MGUS**
Number of patients	1038	58,796
Mean age at index event ± SD	80.4 ± 7.5	78.1 ± 8.5
Sex (%)		
Male	57%	54%
Female	38%	40%
Unknown	5%	6%
Race (%)		
White	83%	84%
Black	8%	4%
Asian	1%	2%
Other / Unknown	8%	10%

Patients with or without monoclonal gammopathy of unknown significance (MGUS, Non-MGUS)

**Fig. 1:**
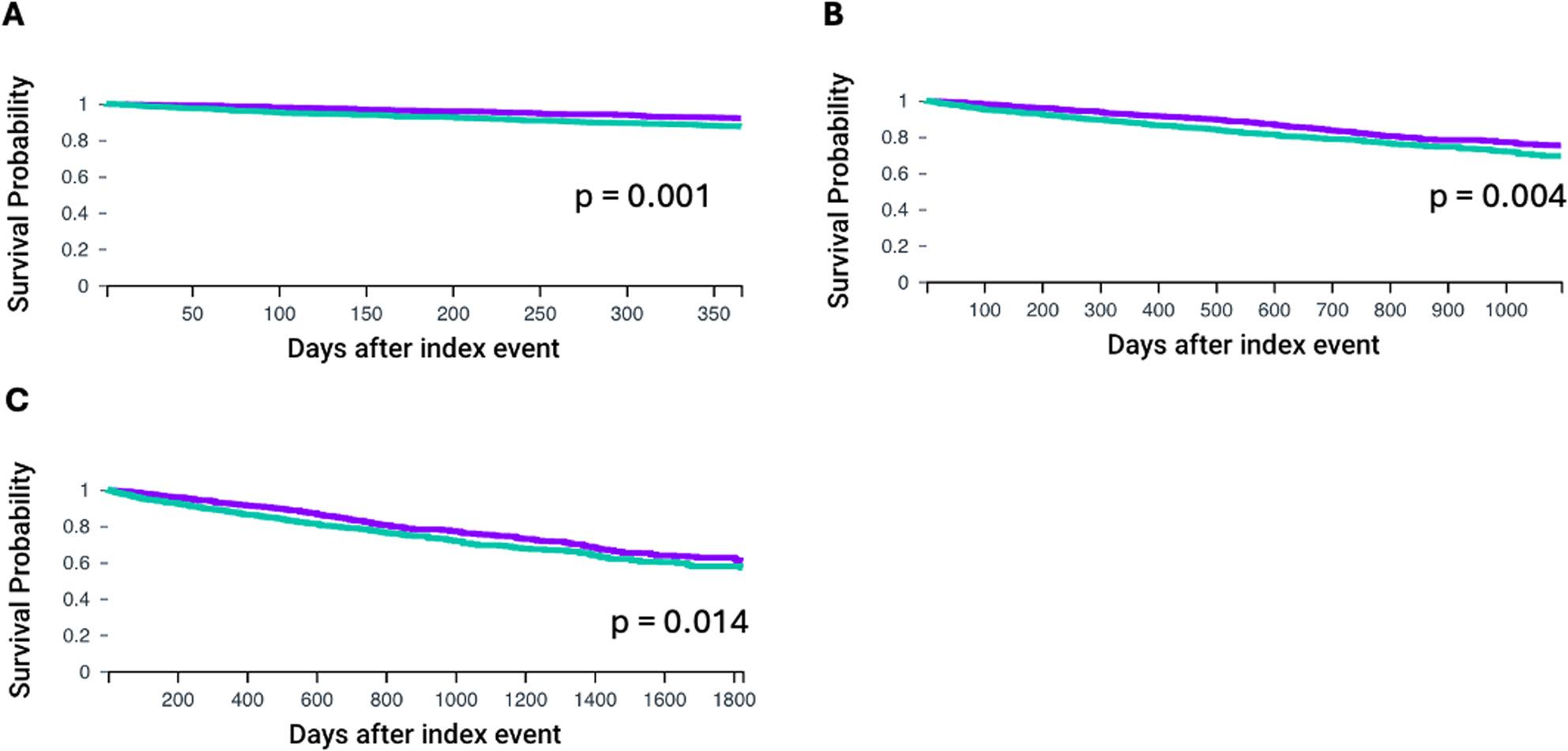
1-, 3-, and 5-year overall survival for patients with or without MGUS after TAVI implantation for non-rheumatic valve stenosis after propensity score matching. **A**: 1-year overall survival, **B**: 3-year overalls survival, **C**: 5-year overall survival. MGUS group is shown with the green line. Propensity matched patients without MGUS are displayed with the purple line (Non-MGUS group). Overall survival was was assessed using Kaplan-Meier analysis. Statistical significance is shown by *p* < 0.05. Monoclonal gammopathy of unknown significance (MGUS), transcatheter aortic valve implantation (TAVI)

First, we compared overall survival for the overall cohort after propensity score matching. TriNetX conducts propensity score matching before every analysis. Thus, the propensity score matched groups may differ slightly in dependence of available HCO informations. A total of 1038 patients were identified with a mean age of approximately 80 years at the time of the index event (78.1 ± 8.5 Non-MGUS and 80.4 ± 7.5 in MGUS). Mean hemoglobin levels (g/dl) were significantly different between the groups (Non-MGUS 11.8 ± 2.1 and MGUS 10.8 ± 2.1), *p* < 0.001). Levels of HbA1c (%) (6.1 ± 1.1 vs. 6.1 ± 1.1, *p* = 0.901) and NT-pro-BNP (pg/ml) did not differ between the groups (Non-MGUS vs. MGUS: 3899 ± 7631.2 vs. 6419.8 ± 8015.7, *p* = 0.134). After propensity sore matching of the respective groups only significant differences in hemoglobin levels remained (Non-MGUS vs. MGUS: 11.6 ± 2.2 vs. 10.8 ± 2.1, *p* < 0.001). When comparing 1-year overall survival patients in the MGUS group had significantly worse outcome (Non-MGUS vs. MGUS, HR: 0.604, CI95% 0.447, 0.816, *p* = 0.001, Fig. 1A). Next, we compared outcomes for 3 and 5 years after the index event. Overall survival was significantly worse in patients with MGUS for both 3- (Non-MGUS vs. MGUS: HR: 0.739, CI95% 0.602, 0.906, *p* = 0.004 Fig. 1B) and 5- year follow-up (Non-MGUS vs. MGUS: HR: 0.792 CI95% 0.657, 0.954, *p* = 0.014 Fig. 1C). All survival curves can be seen in Fig. 1.

**Fig. 2:**
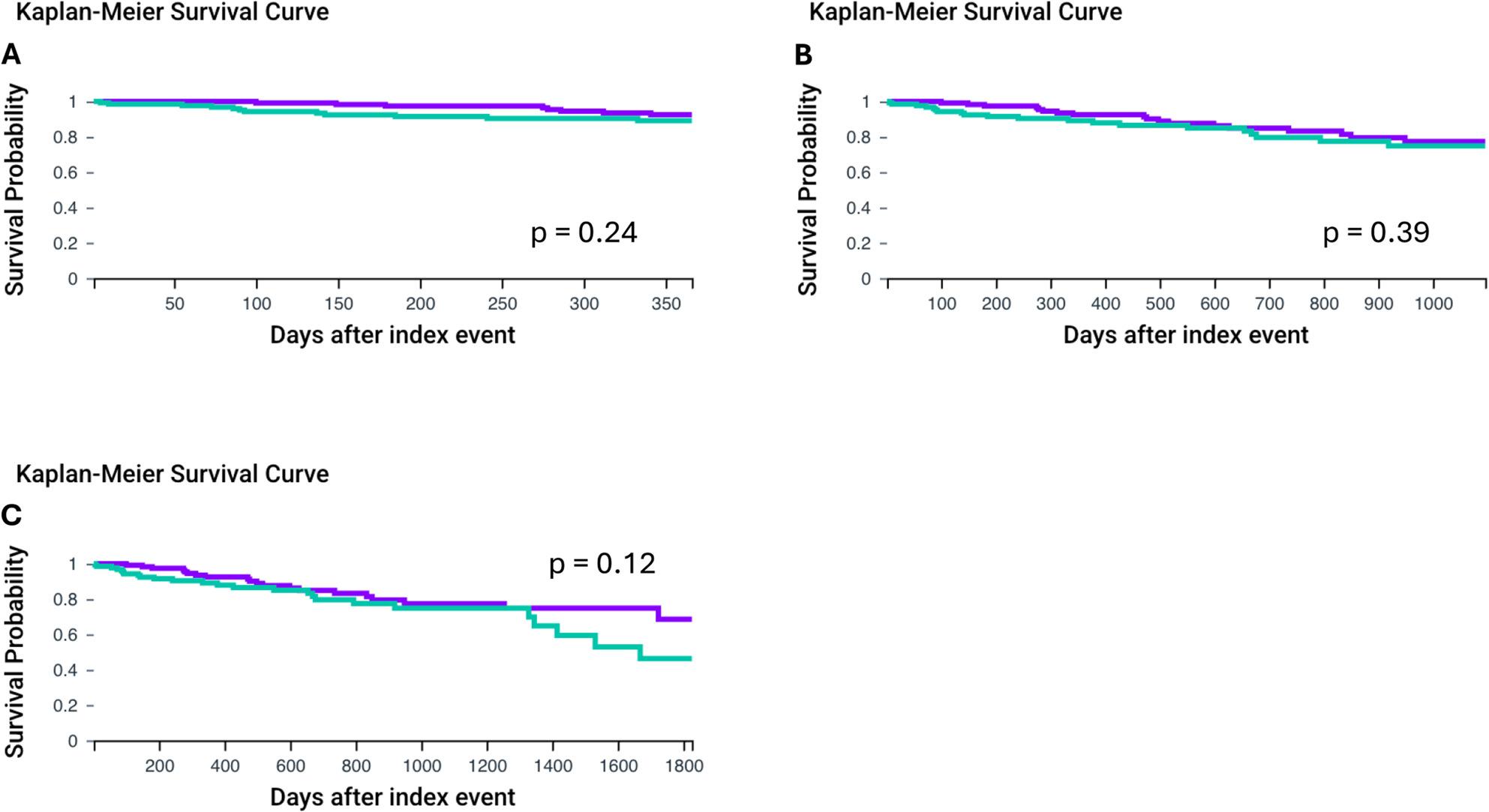
1-, 3-, and 5-year overall survival for patients with or without MGUS after TAVI implantation in patients <75 years of age after propensity score matching. **A**: 1-year overall survival, **B**: 3-year overall survival, **C**: 5-year overall survival. MGUS group is shown with the green line. Propensity matched patients without MGUS are displayed with the purple line (Non-MGUS group). Overall survival was was assessed using Kaplan-Meier analysis. Statistical significance is shown by *p* < 0.05. Monoclonal gammopathy of unknown significance (MGUS), transcatheter aortic valve implantation (TAVI)

Age is an important factor for overall survival in patients undergoing TAVI [16, 17]. We therefore separated our groups in patients older and younger than 75 years of age. First, we compared patients < 75 years of age (*n* = 131). After propensity score matching no differences in the respective matched groups (e.g. diagnosis of neoplasms, NT-pro-BNP, HbA1c) except for hemoglobin levels (Non-MGUS vs. MGUS: 12.5 ± 2.4 vs. 11.7 ± 2.2, *p* = 0.007) remained. Mean age at the index event was 66.7 ± 5.4 for the Non-MGUS and 66.6 ± 5.4 for the MGUS group. Of note only limited numbers of patients had data available for outcome analysis (see Table 3). Overall survival at 1 year was high in both groups with no significant differences (HR: 0.595, CI95%: 0.246, 1.436, *p* = 0.243, Fig. 2A).

**Table 3:** Group comparisons, group sizes and statistical outcomes

Comparison	Patients in Cohort after propensity score matching	Patients with Outcome	Median survival (Days)	Survival Probability at End of Time Window (%)	*p*-value	HR	CI (95%)	Test for proportionality *p* - value
Non-MGUS vs. MGUS: 1-year OS	1038	Non-MGUS: 69	Not reached	Non-MGUS: 92.0	0.001	0.604	0.447, 0.816	0.224
		MGUS: 110		MGUS: 87.4				
Non-MGUS vs. MGUS: 3-year OS	1038	Non-MGUS: 167	Not reached	Non-MGUS: 75.1	0.004	0.739	0.602, 0.906	0.118
		MGUS: 204		MGUS: 69.3				
Non-MGUS vs. MGUS: 5-year OS	1038	Non-MGUS:212	not reached	Non-MGUS: 60.4	0.014	0.792	0.657, 0.954	0.021
		MGUS: 234		MGUS: 57.0				
< 75 years of age								
Non-MGUS vs. MGUS: 1-year OS	131	Non-MGUS: 10	Not reached	Non-MGUS: 92.4	0.248	0.593	0.242, 1.453	0.022
		MGUS: 12		MGUS: 89.1				
Non-MGUS vs. MGUS: 3-year OS	131	Non-MGUS: 18	not reached	Non-MGUS: 77.3	0.397	0.760	0.402, 1.483	0.249
		MGUS: 20		MGUS: 74.8				
Non-MGUS vs. MGUS: 5-year OS	131	Non-MGUS: 20	Non-MGUS: not reached	Non-MGUS: 68.5	0.127	0.634	0.352, 1.145	0.866
		MGUS: 25	MGUS: 1668	MGUS: 46.2				
> 75 Years of age								
Non-MGUS vs. MGUS: 1-year OS	965	Non-MGUS: 86	not reached	Non-MGUS 89.7	0.138	0.805	0.605, 1.072	0.295
		MGUS: 103		MGUS: 87.6				
Non-MGUS vs. MGUS: 3-year OS	965	Non-MGUS: 163	Not reached	Non-MGUS: 75.8	0.008	0.758	0.616, 0.932	0.138
		MGUS: 200		MGUS: 67.6				
Non-MGUS vs. MGUS: 5-year OS	965	Non-MGUS: 206	Not reached	Non-MGUS: 59.9	0.017	0.797	0.660, 0.961	0.673
		MGUS: 232		MGUS: 54.1				
Comorbidities								
COPD								
Non-MGUS vs. MGUS: 5-year OS	68	Non-MGUS: 12	not reached	Non-MGUS: 66.3	0.356	0.700	0.327, 1.498	0.367
		MGUS: 15		MGUS: 66.8				
CKD								
Non-MGUS vs. MGUS: 5-year OS	269	Non-MGUS: 53	not reached	Non-MGUS: 61.1	0.323	0.826	0.565, 1.208	0.537
		MGUS: 54		MGUS: 62.0				
HF								
Non-MGUS vs. MGUS: 5-year OS	189	Non-MGUS: 28	Not reached	Non-MGUS: 73.96	0.079	0.644	0.392, 1.057	0.251
		MGUS: 36		MGUS: 60.03				

*CKD* Chronic kidney disease, *COPD* Chronic obstructive pulmonary disease, *CI* Confidence interval, *HF* Heart failure, *HR* Hazard ratio, *MGUS* Monoclonal gammopathy of unknown significance, *OS* Overall survival

Statistical significance is shown by *p* < 0.05

Furthermore, no significant differences in overall survival were found at the 3-year (Non-MGUS vs. MGUS: HR: 0.760, CI95% 0.402, 1.438, *p* = 0.397, Fig. 2B). and 5-year follow-up (Non-MGUS vs. MGUS: HR: 0.634, CI95% 0.352, 1.145, *p* = 0.127, Fig. 2C).

**Fig. 3:**
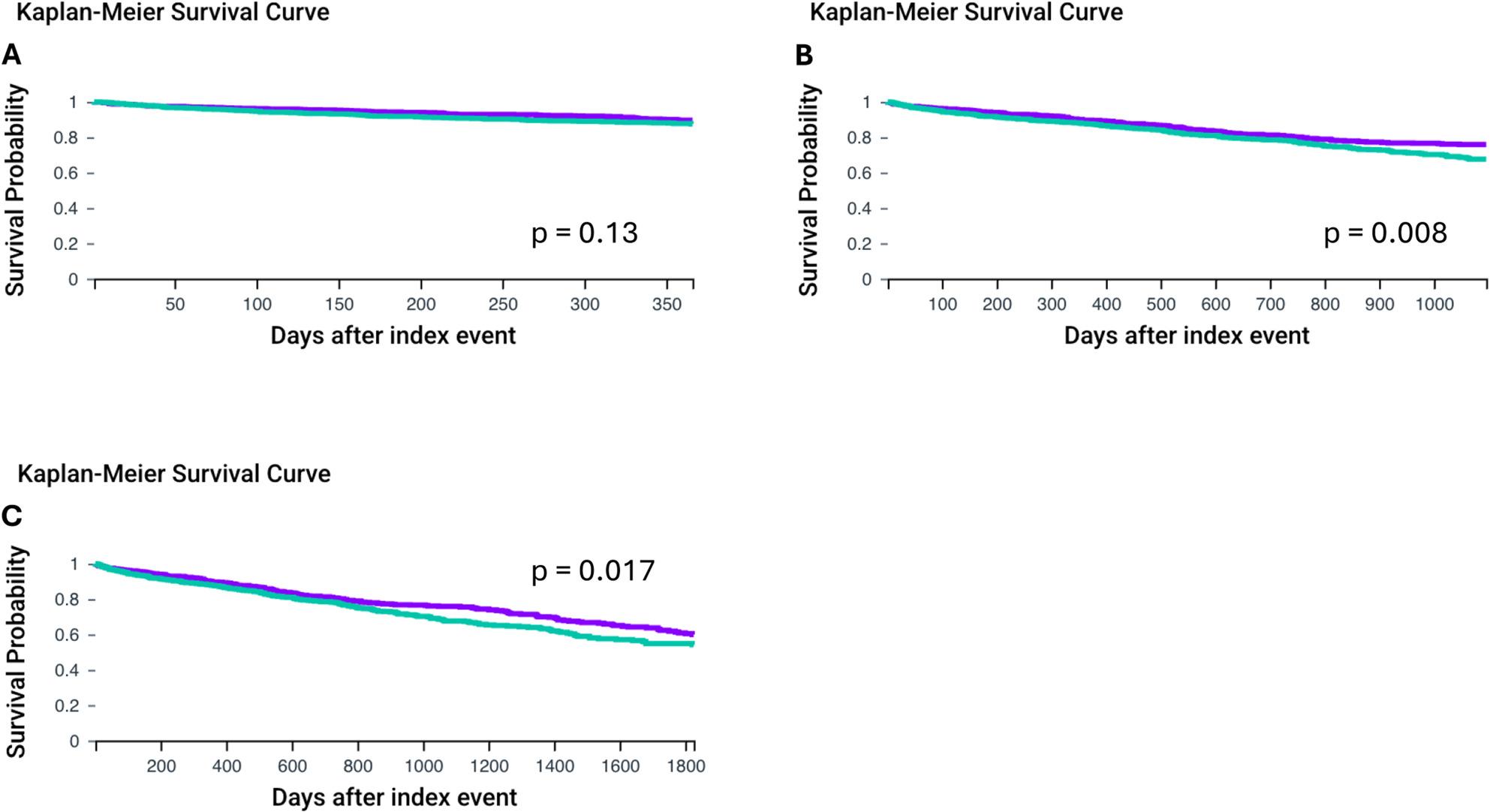
1-, 3-, and 5-year overall survival for patients with or without MGUS after TAVI implantation in patients >75 years of age after propensity score matching. **A**: 1-year overall survival, **B**: 3-year overall survival, **C**: 5-year overall survival. MGUS group is shown with the green line. Propensity matched patients without MGUS are displayed with the purple line (Non-MGUS group). Overall survival was was assessed using Kaplan-Meier analysis. Statistical significance is shown by *p* < 0.05. Monoclonal gammopathy of unknown significance (MGUS), transcatheter aortic valve implantation (TAVI)

Next, we looked at overall survival in patients > 75 years of age. Similar to our previous findings patients with MGUS had lower levels of Hb (Non-MGUS: 11.8 ± 2.2 vs. MGUS: 10.8 ± 2.1, *p* < 0.001) after propensity matching. No differences in levels of NT-pro-BNP and HbA1c were observed. Mean age at the index event was 82.4 ± 5.5 for the group without MGUS vs. 82.3 ± 5.5 with MGUS. 1-year overall survival was similar in both cohorts (Non-MGUS vs. MGUS: HR: 0.805, CI95% 0.605, 1.072, *p* = 0.138, Fig. 3A). In comparison, significantly superior survival was observed in patients without MGUS at 3 years (HR: 0.758, CI95% 0.616, 0.932, *p* = 0.008 Fig. 3B) and 5 years after the index event (HR: 0.797, CI95% 0.660, 0.961, *p* = 0.017, Fig. 3C).

Next, we applied the Cox proportional Hazards model analytic tool of the TriNetX platform to our overall cohort including groups (Non-MGUS vs. MGUS), hemoglobin (< 11 g/dl), age at index and sex as covariates. Group membership to the Non-MGUS group was associated with significantly better outcomes (HR: 0.738, CI95% 0.649, 0.839, *p* < 0.0001) whereas low Hb levels, older age at index event and male sex were associated with inferior overall survival (Hb levels: HR: 2.215, CI95%, 2.105, 2.331, age at index event: HR: 1.026 CI95%, 1.023, 1.028, male: HR: 1.108, CI95%, 1.067, 1.115, all *p* < 0.001).

**Fig. 4:**
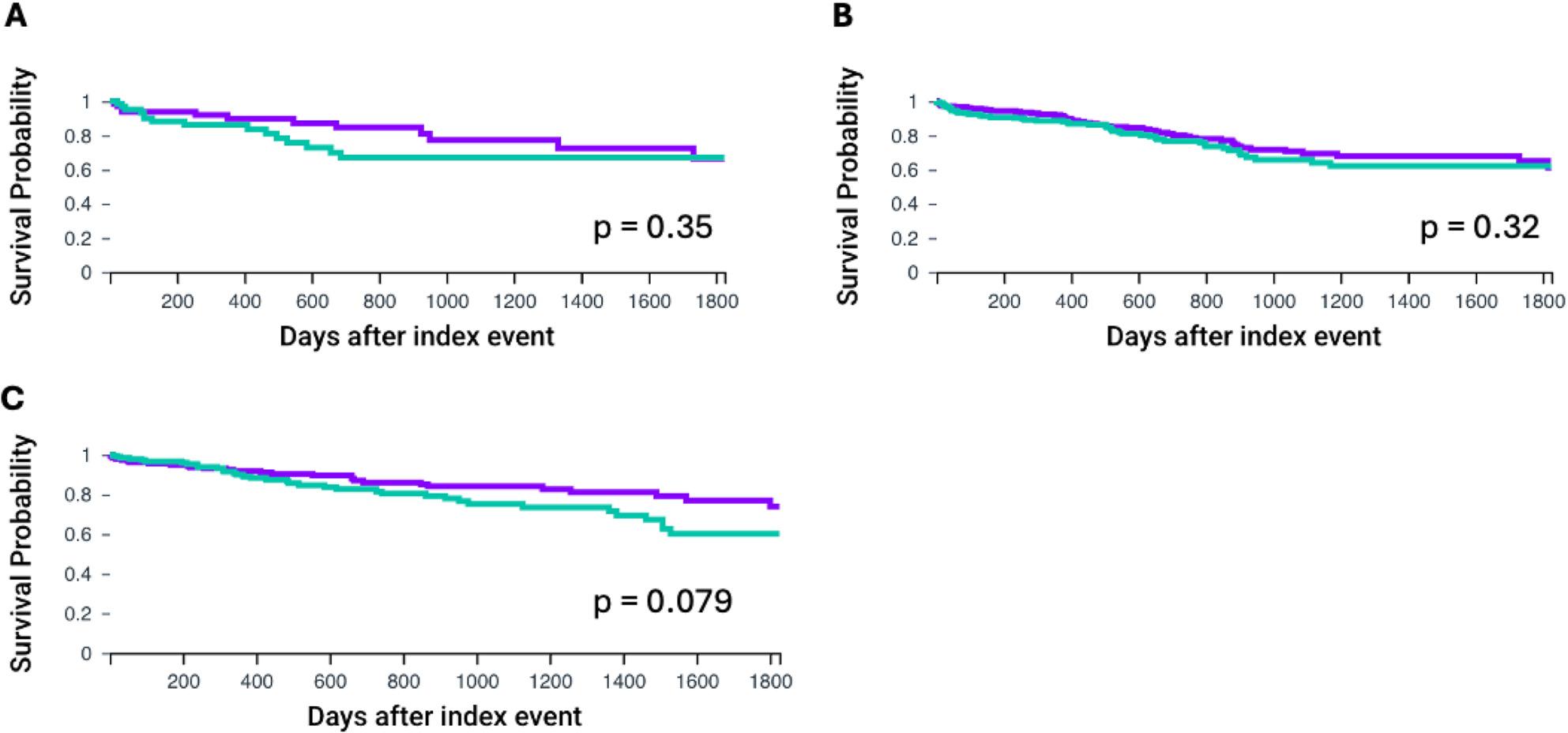
5-year overall survival for patients with or without MGUS after TAVI implantation in the context of propensity score matching. MGUS group is shown with the green line. Propensity matched patients without MGUS are displayed with the purple line (Non-MGUS group). **A**: Chronic obstructive pulmonary disease (COPD), **B**: Chronic kidney disease (CKD), **C**: Heart failure (HF). Overall survival was was assessed using Kaplan-Meier analysis. Statistical significance is shown by *p* < 0.05. Monoclonal gammopathy of unknown significance (MGUS), transcatheter aortic valve implantation (TAVI)

Comorbidities are important risk factors influencing patient quality of life and overall patient survival especially in older patients. Risk scores evaluating perioperative risk in TAVI include pulmonary disease, cardiac performance and renal function [18]. Therefore, we next investigated 5-year overall survival in patients with co-occurring COPD, CKD or HF. After propensity score matching a total of 66 patients were identified with MGUS and co-occurring COPD. Age at the index event was 84.2 ± 6.1 years. Age did not differ significantly in the control group (COPD/Non-MGUS: 83.9 ± 6.2). Overall survival at 5 years did not differ between the groups (HR: 0.7, CI95% 0.327, 1.498, *p* = 0.356, Fig. 4A). Similarly, no differences in overall survival were found for MGUS patients with co-occurring CKD (HR: 0.826, CI95% 0.6565, 1.208, *p* = 0.323, Fig. 4B). A trend to adverse survival rates was observed in patients with MGUS and HF without reaching statistical significance. (HR: 0.644, CI95% 0.392, 1.052, *p* = 0.079, Fig. 4C). All comparisons can be found in Table 3.

## Discussion

Interest in the interactions between cardiovascular diseases and clonal hematological disorders are under growing investigation. CHIP and MGUS are associated with higher incidences of cardiovascular diseases [19]. While the underlying biology is not yet understood, outcomes after TAVI are negatively impacted in patients with CHIP [1, 20]. To our knowledge our study is the first one to date assessing the potential prognostic impact of MGUS in patients having received TAVI. This includes the first analysis of subgroups, answering questions about the relevance of comorbidities, age, the severity of cardiac disease burden and magnitude of hematologic impact caused by MGUS in a real-world data environment.

After propensity score matching most parameters, such as age at index event or sex differences, did not differ between subgroups. However, significantly lower Hb levels remained in the MGUS group in some comparisons. This finding is in line with a recent study in MGUS patients ([21]. Baseline moderate to severe anemia (Hb < 11 g/dl) has been shown to be associated with increased 1-year mortality after TAVI [22]. In our subgroups mean Hb levels below 11 g/dl were observed in the overall and the above 75-year-old MGUS group, possibly influencing outcomes in this cohort. Inferior overall survival for patients with MGUS was shown in the 3-, and 5-year follow-up. Median overall survival has not been reached in both cohorts. In the cox proportional hazard model analysis subgroup membership remained an independent risk factor for overall survival when also considering low levels of Hb as a covariate. We confirmed levels of Hb < 11 g/dl as an independent negative impactor on overall survival in our cohort highlighting the importance of addressing anemia in patients after TAVI. However, associations may change when considering other covariates in statistical models highlighting the need of confirmation of our findings in other patient cohorts.

MGUS has been linked to higher risk for cardiovascular outcome [10, 11]. However mechanistic evidence is missing. In patients with CHIP adverse outcomes after TAVI may possibly be attributed to higher inflammation [23]. Similarly inflammation is altered in MGUS and multiple myeloma [24]. Exemplary high interleukin 6 levels were associated with increased mortality after TAVI in univariate analysis [25] and are elevated in some MGUS patients [26]. Furthermore co-occurrence of MGUS and inflammatory-rheumatic diseases has been shown [27] further suggesting higher inflammation in MGUS patients. While the progression from MGUS to MM as clonal expansion and modifications within the bone marrow niche has been investigated [28–30] further studies are needed to determine the effects of clonal plasmacells on cardiovascular mechanisms.

Age affects outcomes after TAVI and is considered in prognostic risk scores [16, 17, 31, 32]. We first compared overall survival in patients < 75 years of age. Here similar overall survival was found for patients with or without MGUS at 1-, 3-, and 5-year follow-up. However, significant differences in overall survival were found in patients > 75 years at 3 and 5 years with adverse outcomes in the MGUS group. Of note, outcomes were still very good in the older subgroup with the median survival not reached in this vulnerable patient population irrespective of presence of MGUS. Though significantly worse outcomes were found for patients with MGUS, patients remained with a high survival probability at the 5-year follow-up. MGUS is a disease with a median age at diagnosis of 70 years [33] therefore limiting findings and frequency in patients < 75 years of age with a probably higher impact on elderly patients. While we found inferior outcome after TAVI for patients with MGUS in older patients, outcomes did not differ in the younger population at timepoints up to 5 years after procedure. Even though outcomes after TAVI in MGUS patients are good overall, MGUS can be considered as a risk factor for impaired survival in patients older than 75 years of age undergoing TAVI.

Comorbidities are included in risk assessment for TAVI and some studies have shown adverse impact of CKD, COPD and HF after TAVI [34–37]. In our analysis we did not find different overall survival of MGUS patients and the control group in the context of CKD and COPD. Interestingly we found a trend towards adverse outcomes for patients with HF and MGUS after TAVI indicating an aggravating impact of MGUS in HF patients. These findings must be interpreted carefully as available outcome data is limited in these subgroups – especially with COPD - resulting in small sample sizes.

Our study must be interpreted in the context of important limitations associated with the TriNetX platform and its analytical tools and study related limitations due to cohort definition. Given the retrospective nature and lack of detailed cohort information, our results should be interpreted as exploratory and hypothesis-generating rather than causal. TriNetX does not provide individual patient data and genetic information is sparse. The prevalence of MGUS in our cohort was lower (1.76%) than in the general population (3.2% [33]), , reflecting under-coding and/or under-detection which could not be individually followed up due to the lack of individual patient data. Furthermore main predictors for outcome after TAVI, such as STS/EuroScore II ([38, 39], valve type, complications and frailty [40] are not available. Regarding the lack of these variables, our findings should be interpreted as associations rather than causal relationship. We matched our groups for Hb, NT-pro-BNP and HbA1c levels. Anemia and elevated NT-pro-BNP levels have been shown factors for adverse outcome after TAVI [22, 41] while there have been differing results for diabetes mellitus (Type 2) most likely not influencing outcomes [42, 43]. Other group differences and individual comorbidities may influence outcomes and cannot fully be accounted for due to individual data unavailability.

Secondly, outcome data for patients is limited (see Table 3) after propensity score matching, reducing sample sizes in some subgroups. Differences in overall survival, especially in patients < 75 years and comorbidities, may be found with increasing sample sizes. Furthermore, matching is executed before every new analysis by the platforms analytic tool possibly resulting in slightly differing cohorts. Finally, reproducibility is limited as cohort definition depends on response of HCO´s possibly not including the same patient sets. This highlights the need for public worldwide large data sets – especially for rare diseases- to better investigate disease associations of these patients. In this study we created groups according to ICD/CPT codes which are highly influenced by coding of diagnosis and may therefore not completely reflect individual patient reality. Taken together, this underlines the necessity of confirming our findings in different patient cohorts outside of real-world data platforms. Lastly our study investigates overall survival which can be impacted by comorbidities in an older study population. For example, adverse outcome after TAVI in COPD patients have been linked to higher exacerbation rates instead of cardiac associated mortality [44]. Future studies should aim to differentiate cardiac associated mortality after TAVI in MGUS patients. Furthermore, even though TriNetX is a global network most HCO were located in the United States of America and Europe limiting these results to these regions. Lastly certain misrepresentations could not be adjusted for, such as underrepresentation of minority groups in the respective cohorts.

Summarizing, our results show inferior overall survival for patients with MGUS in patients older than 75 years of age. Long-term overall survival did not differ in older patients with COPD and CKD but tended to be inferior in those with HF. Therefore, our data, in consideration of the above mentioned important study limitations suggest an aggravating impact of MGUS in elderly patients with HF undergoing TAVI. While further studies are needed to confirm these findings and explore the underlying biological mechanisms, this study contributes to the rising field of cardio-oncology, providing the hypothesis of MGUS impacting mortality following TAVI in elderly patients.

This study may provide first rationale to further investigate the impact of monoclonal gammopathy on cardiovascular disease and highlights the need for interdisciplinary research to improve outcome prediction and patient care.

## Data Availability

All data can be obtained by request from the corresponding author ( [ingo.eitel@uksh.de](mailto: ingo.eitel@uksh.de) **). Individual patient date is not available due to TriNetX data protection.**.

## Abbreviations

AL: Amyloidosis
CHIP: Clonal Haematopoiesis of Unknown Significance
CI: Confidence interval
CKD: Chronic Kidney Disease
COPD: Chronic Obstructive Pulmonary Disease
CPT: Current Procedural Terminology
EMR: Electronic medical record
Hb: Hemoglobin
HbA1c: Hemoglobin A1c
HCO: Health care organisation
HF: Heart Failure
HIPAA: US Health Insurance Portability and Accountability Act
HR: Hazard ratio
ICD: International classification of diseases
MGUS: Monoclonal Gammopathy of Unknown Significance
MM: Multiple myeloma
NrVS: Non-rheumatic aortic valve stenosis
NT-pro-BNP: N-terminal prohormone of brain natriuretic peptide
TAVI: Transcatheter Aortic Valve Implantation
